# Parabolic gratings enhance the X-ray sensitivity of Talbot interferograms

**DOI:** 10.1038/s41598-023-36414-8

**Published:** 2023-06-27

**Authors:** Pouria Zangi, Katsumasa Ikematsu, Pascal Meyer, Hidekazu Takano, Yanlin Wu, Josephine Gutekunst, Martin Börner, Arndt Last, Jan G. Korvink, Atsushi Momose

**Affiliations:** 1 Institute of Microstructure Technology, Karlsruhe Institute of Technology, Hermann-von-Helmholtz-Platz 1, 76344 Eggenstein-Leopoldshafen, Germany; 2 Institute of Multidisciplinary Research for Advanced Materials, Tohoku University, 2-1-1 Katahira, Aoba-ku, Sendai, Miyagi 980-8577 Japan; 3 Karlsruhe Nano Micro Facility (KNMFi), Karlsruhe Institute of Technology, Hermann-von-Helmholtz-Platz 1, 76344 Eggenstein-Leopoldshafen, Germany

**Keywords:** Engineering, Materials science, Optics and photonics

## Abstract

In grating-based X-ray Talbot interferometry, the wave nature of X-ray radiation is exploited to generate phase contrast images of objects that do not generate sufficient contrast in conventional X-ray imaging relying on X-ray absorption. The phase sensitivity of this interferometric technique is proportional to the interferometer length and inversely proportional to the period of gratings. However, the limited spatial coherency of X-rays limits the maximum interferometer length, and the ability to obtain smaller-period gratings is limited by the manufacturing process. Here, we propose a new optical configuration that employs a combination of a converging parabolic micro-lens array and a diverging micro-lens array, instead of a binary phase grating. Without changing the grating period or the interferometer length, the phase signal is enhanced because the beam deflection by a sample is amplified through the array of converging-diverging micro-lens pairs. We demonstrate that the differential phase signal detected by our proposed set-up is twice that of a Talbot interferometer, using the same binary absorption grating, and with the same overall inter-grating distance.

## Introduction

Since the first X-ray image was taken in 1895^1^, X-ray absorption-contrast imaging became a standard tool for diagnostic medical imaging and non-destructive testing. Despite its wide spread use, the generated contrast in X-ray absorption imaging is low for weakly absorbing materials, e.g., materials with low atomic Z numbers^2^. The experimental realization of X-ray phase-contrast imaging^3^, provided complementary information to the absorption contrast, based on the fact that the atomic interaction cross section of phase shift is about a thousand times larger than the absorption cross section.

X-ray phase imaging methods are categorized into: two-beam interferometry^3^, diffraction-enhanced imaging^4^, propagation-based imaging^5^, coherent diffraction imaging^6^, and grating interferometry^7,8^. Among those, X-ray phase imaging with grating interferometry, more specifically X-ray Talbot interferometry, has attracted attention because of its flexibility in usage even with a laboratory X-ray source. It utilizes the self-imaging effect (or fractional Talbot effect) generated by a phase grating (referred to as G1). When a sample is placed in front of or behind G1, the self-image is laterally displaced because of the refraction of the sample. The displacement is analyzed by the second grating, which is an absorption grating (referred to as G2) and placed at the position of the self-image with an especially high visibility that is termed the condition of the fractional Talbot effect. The image detector, placed behind G2, records moiré images, which are the result of the superposition of the self-image and the transmission function of G2.

The sensitivity of the Talbot interference to the refraction is inversely proportional to the grating’s period. However, since a high aspect-ratio structure is needed especially for G2, grating fabrication capabilities limit the smallest achievable period^9,10^. The phase signal is also proportional to the distance between G1 and G2, since the displacement of the self-image is proportional to the inter-grating distance. However, increasing the inter grating distance demands higher transverse coherence from illuminating X-rays^8^. In addition, the geometrical constraints in compact set-ups limit the maximum inter-grating distance.

Here, we propose an optical arrangement that uses converging and diverging parabolic micro-lens arrays instead of a binary grating (G1) in a Talbot interferometric set-up. The aim is to introduce a mechanism for enhancing the sensitivity of Talbot interferometry without either reducing the grating period, or increasing the inter-grating distance. In the set-up, a converging concave parabolic micro-lens array (L1) is placed behind the sample of interest, and a diverging convex parabolic micro-lens array (L2) is positioned within the focal length of L1. The function of the combination of L1 and L2 corresponds to G1 in a Talbot interferometer, but in addition, the beam deflection angle caused by the refraction at the sample is amplified. The self-image with the amplified lateral shift is analyzed by G2 in the same manner as that of a Talbot interferometer.

To perform an experiment that demonstrates the sensitivity enhancement, L1 and L2 transmission parabolic gratings (i.e., periodic parabolic phase modulation) with a period of 10 μm were designed for 17 keV (0.72 Å) X-rays and were fabricated using a deep X-ray lithography process, which offers the needed optical quality side wall surfaces. A synchrotron radiation experiment was performed at the BL20XU beamline of SPring-8, Japan to show the agreement with simulation, and the enhancement of the differential phase signal. In this paper, the details of the sensitivity enhancement idea and the experimental demonstration results are described.

## Optical configuration concept

**Figure 1:**
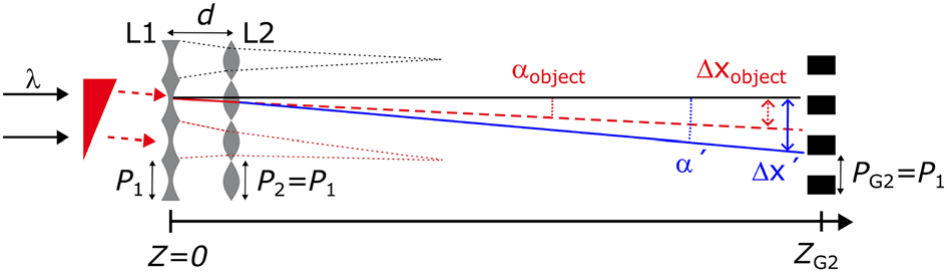
Set-up for sensitivity-enhanced X-ray phase imaging. A combination of a converging (L1) and a diverging (L2) parabolic micro-lens array, separated by *d*, is employed at the G1 position of a conventional Talbot interferometer, replacing its binary phase grating. See text for an explanation of the other variables used in the figure.

We consider a combination of converging (L1) and diverging (L2) parabolic micro-lens arrays used as grating G1, as shown in Fig. 1, whereas a binary phase grating is employed as G1 in a conventional Talbot interferometer.

We assume that L1 and L2 have the same period $P_1=P_2=P$, their focal lengths being $f_1$ and $f_2$, respectively, and that the lenses are separated by $d<f_1$. When a phase object is positioned upstream of L1, the incoming beam is refracted by the angle $\alpha _\mathrm{{object}}$. If a single binary grating, or only L1, is used, $\alpha _\mathrm{{object}}$ results in a lateral shift $\Delta x_\mathrm{{object}}$ of the self-image at G2 that is positioned at a distance of $Z_\mathrm{{G2}}$ from L1. However, due to the presence of the diverging micro-lens array L2, the incoming beam is further deflected, and therefore the self-image lateral shift at G2 should be $\Delta x^{\prime }$ instead of $\Delta x_\mathrm{{object}}$. Here, we define the self-image lateral shift enhancement factor, *M*, which is derived by straightforward geometrical considerations as

1 $$\begin{aligned} M = \frac{\Delta x^{\prime }}{\Delta x_\mathrm{{object}}} = \frac{\alpha ^{\prime }}{\alpha _\mathrm{{object}}}\frac{Z_\mathrm{{G2}}-d}{Z_\mathrm{{G2}}} + \frac{d}{Z_\mathrm{{G2}}}, \end{aligned}$$

where

2 $$\begin{aligned} \frac{\alpha ^{\prime }}{\alpha _\mathrm{{object}}} = 1 - \frac{d}{f_2}. \end{aligned}$$

Equation (2) is obtained by assuming that the lenslets of L1 and L2 have common primary rays, and that $\textrm{tan}(\alpha _\mathrm{{object}}) < P_2/d$. It is implied that choosing a diverging micro-lens array for L2, or in other words a negative $f_2$, results in an enhancement factor $M>1$.

## Simulation

**Figure 2:**
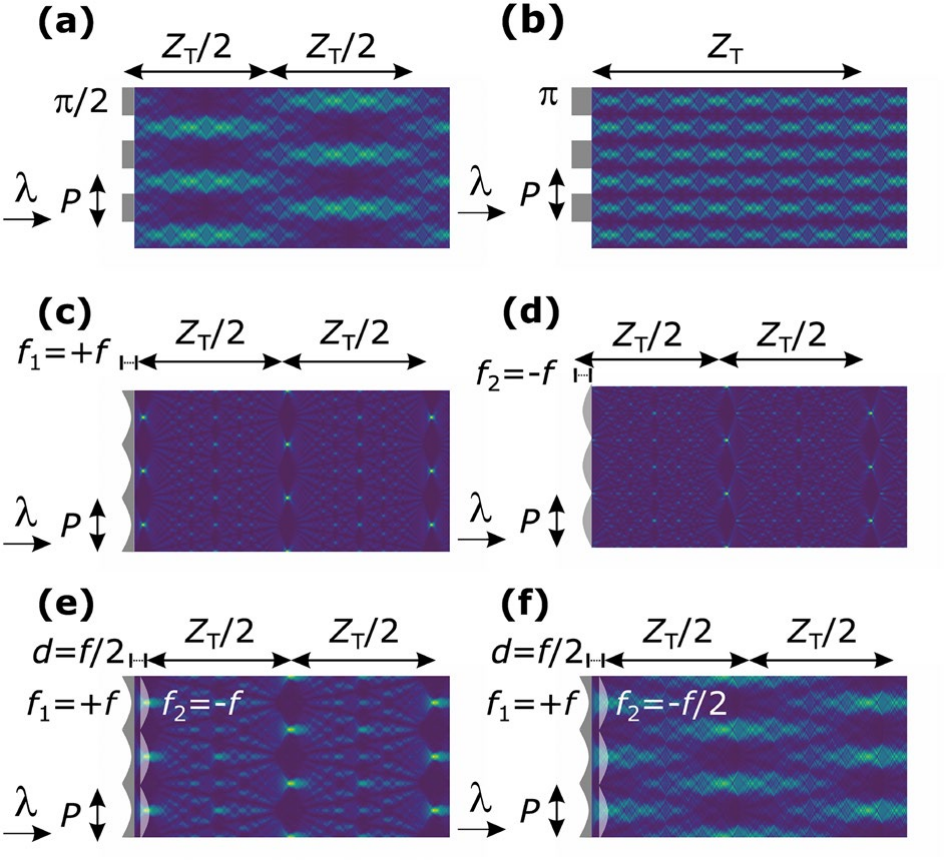
Wave field simulation results. Insets (**a**) and (**b**): downstream of conventional binary phase gratings with the phase modulation of $\pi /2$ and $\pi$, respectively. Insets (**c**) and (**d**): downstream of a converging parabolic grating (L1) and a diverging parabolic grating (L2), respectively. Inset (**e**): downstream of an L1–L2 lens pair separated by $d=f/2$, assuming $f_1 = - f_2 = f$. Inset (**f**): downstream of L1–L2, when only the focal length of L2 is reduced to *f*/2. Here, $f=Z_\mathrm{{T}}/32$.

We performed a simulation study to clarify the intensity profile of the wave field formed by the L1–L2 lens pair in comparison with the wave field formed by a binary grating, assuming monochromatic illumination. The wave field behind a transmission grating can be calculated by the convolution of the grating complex amplitude transmission function, and the Fresnel transfer function^11,12^. Figure 2a,b show the simulated wave fields behind a binary phase grating, with a phase modulation of $\pi /2$ and $\pi$, respectively, where ${Z_T}$ is the so-called Talbot distance under plane wave illumination given by

3 $${Z_T} = 2\frac{{{P^2}}}{\lambda}.$$

Here $\lambda$ is the incoming beam wavelength^13^. At specific positions from the grating, a self-imaging effect (that is, the appearance of high-contrast regions) is observed. This effect can also be noticed for converging and diverging micro-lens arrays^14,15^. Figure 2c,d show the simulation results when the focal lengths of the micro-lens array elements are assumed to be $f=Z_\mathrm{{T}}/32$. In this case, prominent focusing spots are seen in the wave fields. The spots appeared at positions corresponding to multiples of ${Z_T}/2 + f$ for the converging and ${Z_T}/2-f$ for the diverging lens arrays, whereas the self-imaging of a $\pi /2$ binary phase grating appears at positions corresponding to odd multiples of ${Z_T}/4$.

To simulate the wave field downstream of the L1–L2 lens pair, a cascading optical system^16^ was considered; that is, the resulting wave field from L1 at the position of L2 was taken as input for the calculation of the wave field downstream of L2. The simulated wave field downstream of L1–L2 is shown in Fig. 2e, for which the focal lengths of L1 and L2 were the same as $Z_\mathrm{{T}}/32$ and $d = Z_\mathrm{{T}}/64$. The self-imaging feature is similar to that of Fig. 2c or d. However, when the focal length of L2 is halved, the wave field varies considerably, as shown in Fig. 2f.

In Talbot interferometry, the lateral shift of self-images from G1 is used to extract the differential phase signal from a sample. G2 is placed at the position of the self-image to analyze its deformation, which is recorded by a procedure referred to as phase stepping^17^. For this, the period of G2 is selected to be comparable to that of the self-image. G2 or G1 is then moved laterally and parallel to the grating’s periodic pattern by a fraction of its period, and the beam intensities right behind G2 are registered for each step, to obtain the so-called phase stepping curve at each detector pixel position. The required phase signal is extracted by comparing the phase stepping curves obtained with and without a sample^7^. In the following, we outline the conditions under which the use of an L1–L2 lens pair results in a wave field suitable for the phase stepping procedure, G2 being the binary grating.

In Talbot interferometry, the fundamental component of the Fourier expansion series of the phase stepping curves is used for phase imaging^7,8^. Therefore, in addition to the wave field simulation (such as those shown in Fig. 2), phase stepping curves were simulated by assuming the use of a binary absorption grating G2 with a period of *P* and a duty cycle (ratio of the lamellae width to its period) of 0.5, for which the fundamental (or sinusoidal) component of the phase stepping curve was extracted. In line with the condition of the experiments described later, the focal length for L1 was set to $f_1=Z_\mathrm{{T}}/32$.

From the phase stepping curve, $\xi$ given by

4 $$\begin{aligned} \xi = 2 \frac{\Re [a_1]}{a_0} \end{aligned}$$

is evaluated. Here, $a_0$ is the 0th Fourier component of the stepping curve, and $a_1$ is the 1st component whose spatial frequency is $2\pi /P$. $\Re [~]$ extracts the real part of its argument. The visibility of the stepping curve nearly corresponds to $|\xi |$, because higher-order Fourier components are not normally prominent. Note that $\xi$ is negative when the self-image contrast is inverted.

**Figure 3:**
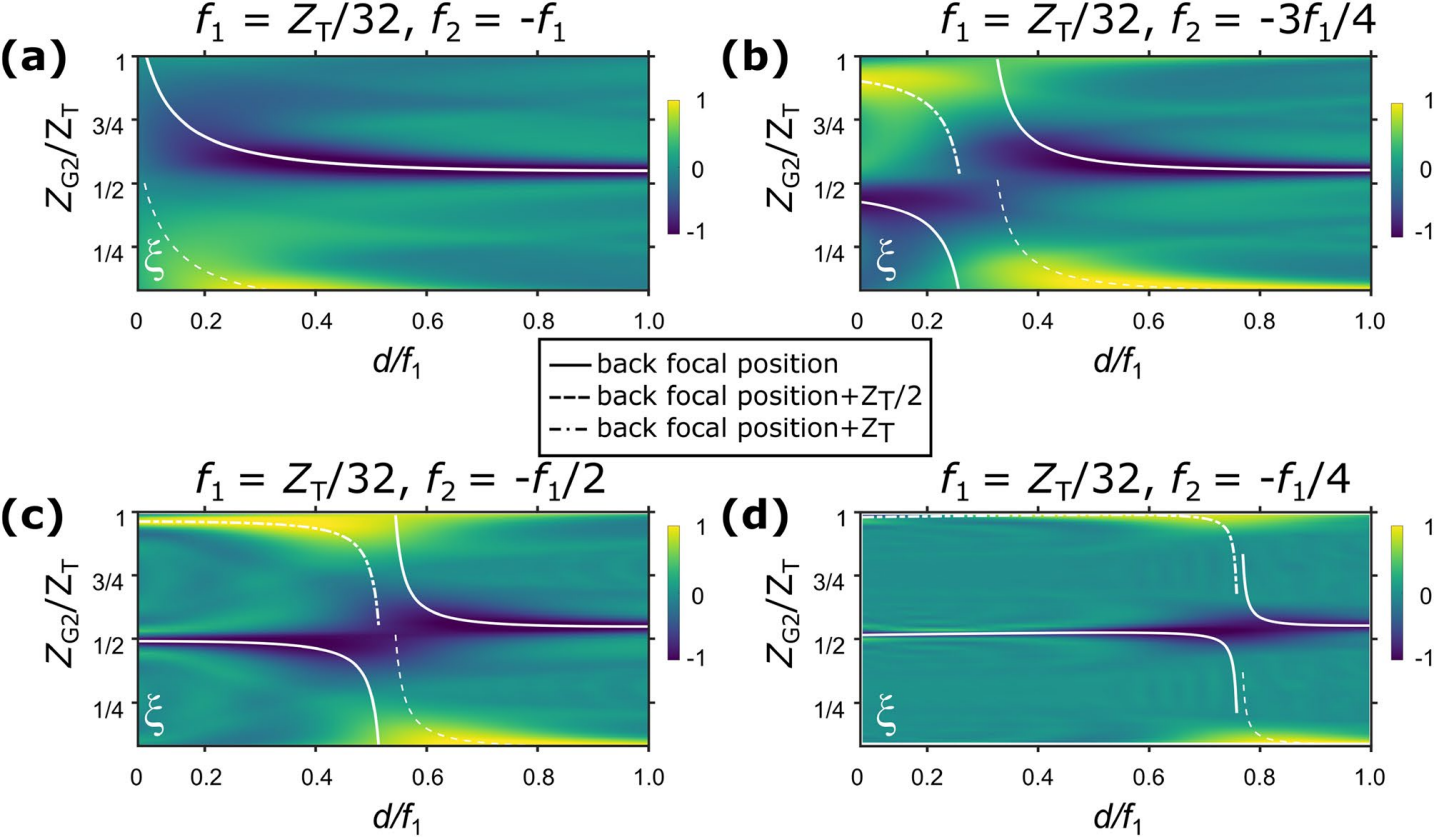
Calculated $\xi$ values corresponding to a binary absorption grating with period equal to that of the parabolic grating, and a duty cycle of 0.5. From (**a**) to (**d**), $f_2$ is set equal to $mf_2/4$, ordered by $m\in \{1,2,3,4\}$, assuming $f_1 = Z_T/32$. The dashed lines indicate the rear focal positions of the L1–L2 lens pair, and solid and dot-dashed lines indicate the positions further downstream from the rear focal position by $Z_\mathrm{{T}}/2$ and $Z_\mathrm{{T}}$, respectively.

The results of the simulated $\xi$ are shown in Fig. 3a,b,c,d for $f_2 =- m f_1/4$ with $m \in \{ 1, 2, 3, 4\}$, respectively, as functions of $d/f_1$ and $Z_\mathrm{{G2}}/Z_\mathrm{{T}}$. The dashed lines indicate the back focal position of the L1–L2 lens pair calculated by the geometrical optics for thin lenses^18^. In addition, solid and dot-dashed lines indicate the positions further downstream of back focal position by $Z_\mathrm{{T}}/2$ and $Z_\mathrm{{T}}$, respectively. This simulation results demonstrate that the G2 positions suitable for obtaining a high-visibility stepping curve varies considerably, depending on the selection of *d* and the focal length.

**Figure 4:**
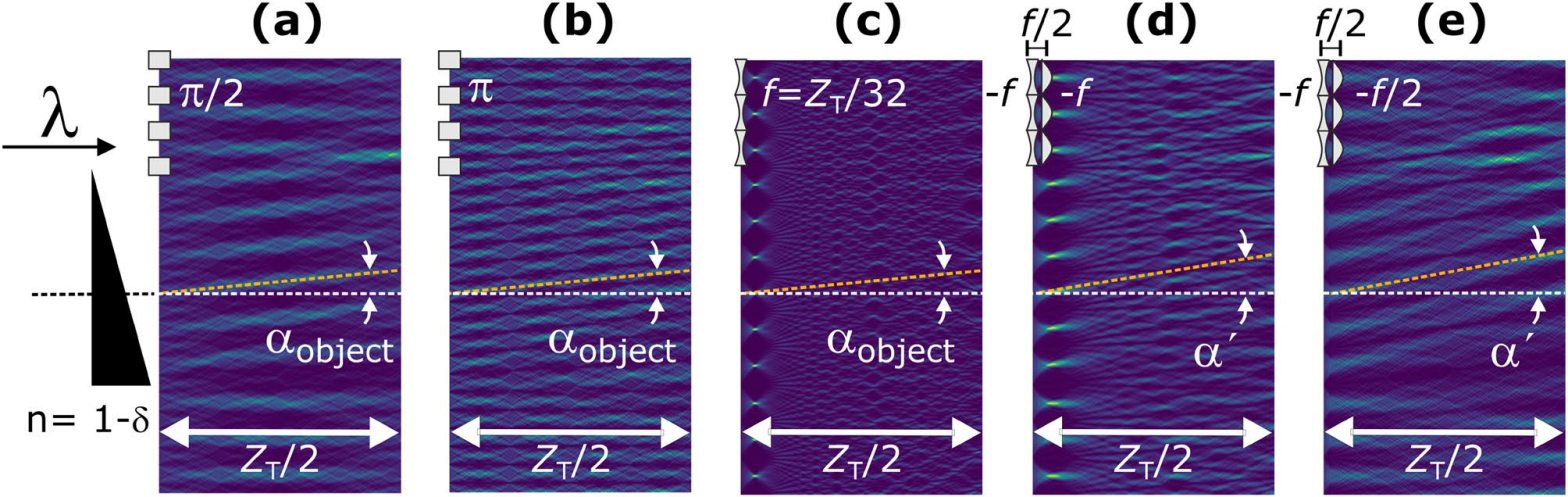
Wave fields in the presence of a wedge-shaped object that introduces the beam refraction of $\alpha _\mathrm{{object}}$ simulated with (**a**) a $\pi /2$ and (**b**) a $\pi$ binary phase grating, (**c**) a converging parabolic gratings with the focal length of $Z_\mathrm{{T}}/32$, the combination of converging and diverging parabolic gratings of the same focal lengths of *f* and with the separation of (d) $d= f$ and (e) $d= f/2$. ${\alpha ^{\prime }}$ is the beam deflection in (**d**) and (**e**), which is larger than that of incoming beam, $\alpha _\mathrm{{object}}$.

Next, we simulated the wave field variations for a wedge-shaped phase object placed in front of a $\pi /2$ binary phase grating (Fig. 4a), a $\pi$ binary phase grating (Fig. 4b), a concave parabolic phase grating (Fig. 4c), and an L1–L2 lens pair (Fig. 4d,e). The wedge-shaped object introduces a beam refraction of $\alpha _\mathrm{{object}}$, and the wave fields are correspondingly deflected. Whereas the wave fields downstream of single gratings, regardless of their shape, are inclined by $\alpha _\mathrm{{object}}$ as shown in Fig. 4a,b,c, it is shown that the wave fields downstream of the L1–L2 lens pair are further inclined, clearly suggesting the sensitivity enhancement effect.

## Experiment

### Parabolic grating fabrication

**Figure 5:**
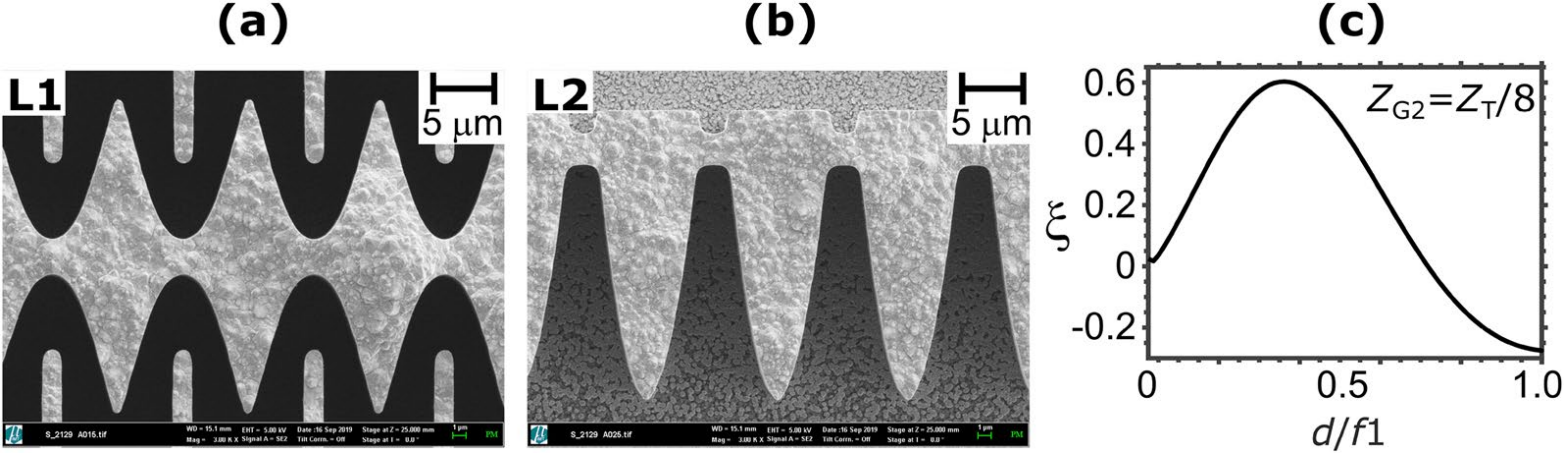
Scanning electron microscope (SEM) images of the fabricated biconcave parabolic grating (**a**) and plano-convex parabolic grating (**b**). The comb-shaped support structures in (**a**) were used to minimize the swelling during the electroplating step and were removed when used. Using the micro-lens array profiles observed in (**a**) and (**b**), the $\xi$ is calculated as a function of $d/f_1$, as shown in (**c**).

For the set of L1 and L2, biconcave and plano-convex micro-lens arrays were fabricated at the Karlsruhe Research Accelerator (KARA) and at the Institute of Microstructure Technology (IMT) using the deep X-ray LIGA process. LIGA is a German acronym for Lithographie, Galvanoformung, Abformung^19^. The plano-convex and biconcave parabolic groove cross-section gratings are in nickel with a designed period of 10 μm, a physical aperture of 9 μm, and a height of 60 μm. The designed parabola’s radius of curvature was 0.429 $\mu$m for each plano-convex and 0.858 μm for each concave parabolic curve, resulting in an intended focal length of 69.4 mm at an energy of 17 keV. Scanning electron microscope (SEM) images of the biconcave and plano-convex micro-lens arrays are presented in Fig. 5a,b, respectively. The focal length and focal size of the biconcave lens at a photon energy of 17 keV were measured to be 77 mm and 0.56 μm (FWHM) by a knife edge scan at the BL20XU beamline of SPring-8, Japan. The measured focal length was in good agreement with calculation from the parabola’s contours as obtained from SEM images (80 mm). Regarding the plano-convex micro-lens array, although X-ray measurements were not performed, the SEM images suggested a focal length of -62.8 mm (see Supplementary Note 1, Supplementary Figure 1, and Supplementary Figure 2). The slight difference between the designed and measured focal lengths can be attributed to the swelling during electroplating of the PMMA photo-resist used for the fabrication of the micro-lens arrays, which caused the radius of curvature of the biconcave parabolas to increase and, *de facto*, decrease that of the plano-convex parabolas.

In Figs. 2 and 3, simulation results are presented for the case of $f_1 = Z_T/32=86$ mm. As mentioned before, the measured focal length of the fabricated biconcave micro-lens array was close to this value.

Figure 5c shows $\xi$ simulated for the fabricated concave plano-convex lenses with G2 positioned at $Z_\mathrm{{T}}/8$, suggesting that $d/f_1=0.35$ would be the most suitable value. Because of our experimental constraint, we chose $d/f_1=0.5$. A reasonably acceptable value of $\xi =0.46$ is attained for the proof-of-concept experiment described later.

To compare the biconcave plano-convex system with a conventional Talbot interferometer using binary gratings, a nickel binary phase grating with the period of 10 μm, duty cycle of 0.5, and phase modulation of $\pi /2$ at a photon energy of 17 keV, was prepared. In addition, an absorption grating with a period of 10 μm, duty cycle of 0.5, and gold lamellae height of 25 μm, was fabricated.

### Wave field measurement

**Figure 6:**
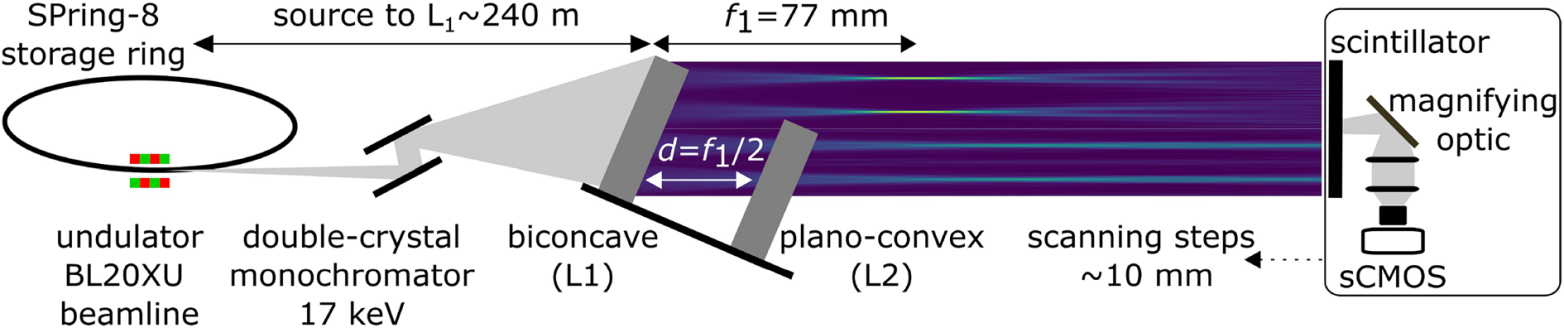
The experimental set-up used in this study at the BL20XU beamline, SPring-8, Japan. A monochromatic incoming beam with an energy of 17 keV reaches the gratings located at a position 240 m from the undulator source. The intensities were detected using a CCD camera coupled with a scintillator and magnifying optic. The detector was mounted on a moving stage to measure the wave field along the beam propagation direction.

The wave field downstream of the fabricated L1 and L2 lenses was evaluated with 17-keV X-rays at BL20XU beamline of SPring-8, Japan. To separately measure the wave fields passing through L1 only, and through both L1 and L2, the concave and plano-convex lenses, placed on a common plate, were tilted slightly against the beam direction (around 0.02°) as shown in Fig. 6. By this, it was possible to simultaneously measure the wave fields formed after passing only L1 (upper beam section) and through the biconcave and L2 (lower part beam section). The wave field intensities are registered by a sCMOS camera (Hamamatsu C11440-22CU) coupled with lens system (Hamamatsu AA50) and scintillator (LuAG(Ce) 10 μm in thickness).

**Figure 7:**
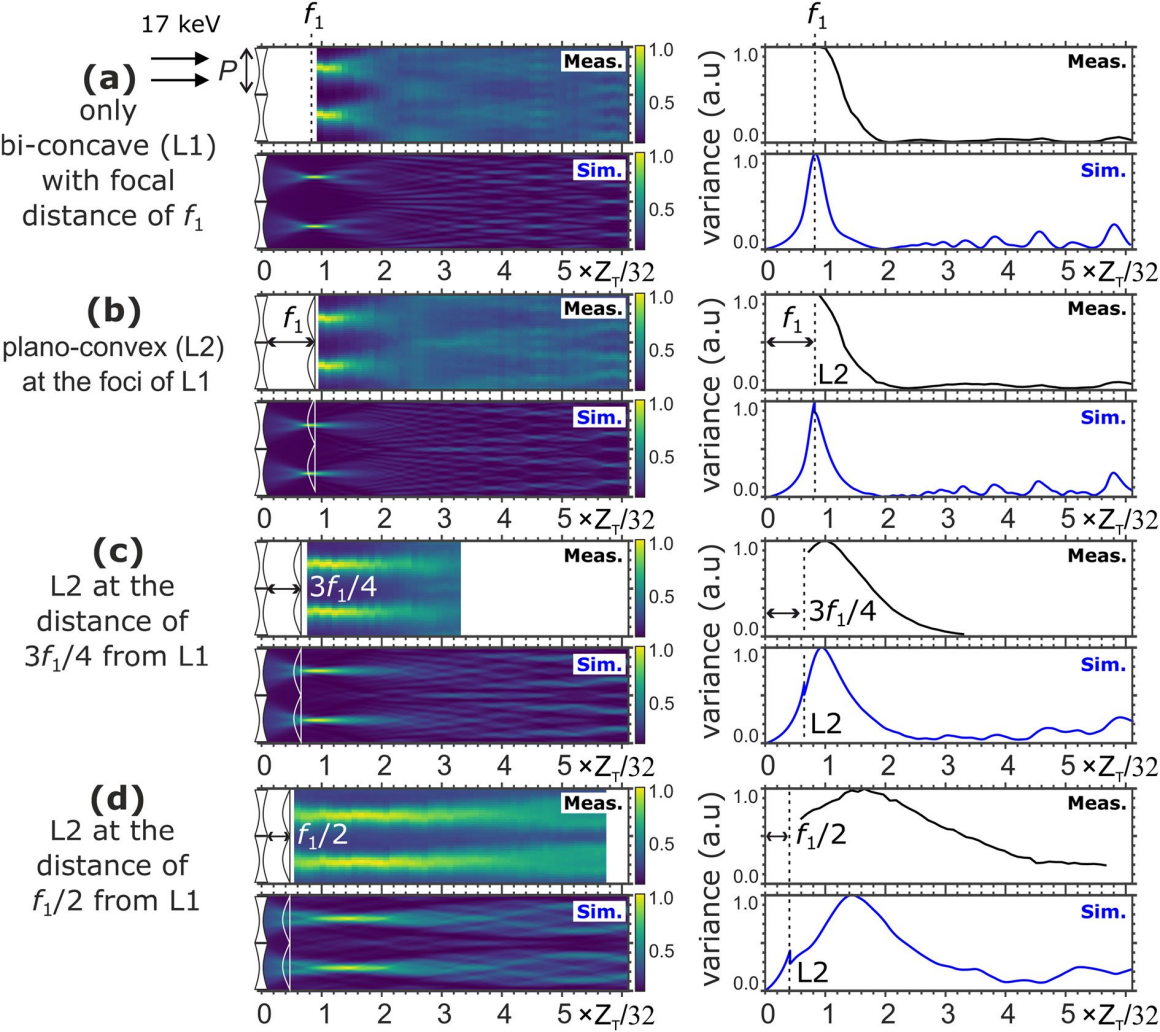
The simulated (Sim.) and measured (Meas.) wave fields downstream of the lens arrays at 17 keV, and the corresponding intensity variances along the center line of a lens. The results are presented (**a**) for only the biconcave lenses, (**b**) plano-convex at the foci $f_1$ of the biconcave, (**c**) plano-convex and biconcave gap of $3f_1/4$, and (**d**) plano-convex at the half distance of the biconcave foci. In (**c**), the measured wave fields downstream could only be performed up to the propagation distance of Z= 310 mm. A white color in the measured wave fields indicates that no measurement was made at that point.

The measured and simulated wave fields and corresponding intensity variance are shown in Figs. 7a,b,c,d for the cases of L1 only and different L1–L2 distances. These simulation results were obtained using the fabricated parabola’s contours extracted from the SEM images shown in Fig. 5.

The features observed in the measured wave fields are in good agreement with simulation results, except for the fact that the measured wave fields tended to be spread slightly. We speculate that the deviation was caused by the limited spatial coherency of the incoming beam, and imperfectness of the fabricated gratings. However, there was no concern when considering that G2 will be placed for generating differential phase contrast. It is worth noting that a highly contrasted wave field was formed over a larger extent behind the L1–L2 position, set by tuning the L1–L2 distance. When $d/f_1 = 0.5$, as shown in Fig. 7d, this effect was prominent, suggesting a wide tolerance in selecting the position of G2. Figure 7d indicates a proper position at $Z_T/8$, provided that G2 with a duty cycle of 0.5 is used. Therefore, we chose an L1–L2 gap of $f_1/2$ and at $Z_T/8$, for *G*2 positioned to demonstrate phase imaging, as shown in the next section.

### Demonstration of sensitivity enhancement

**Figure 8:**
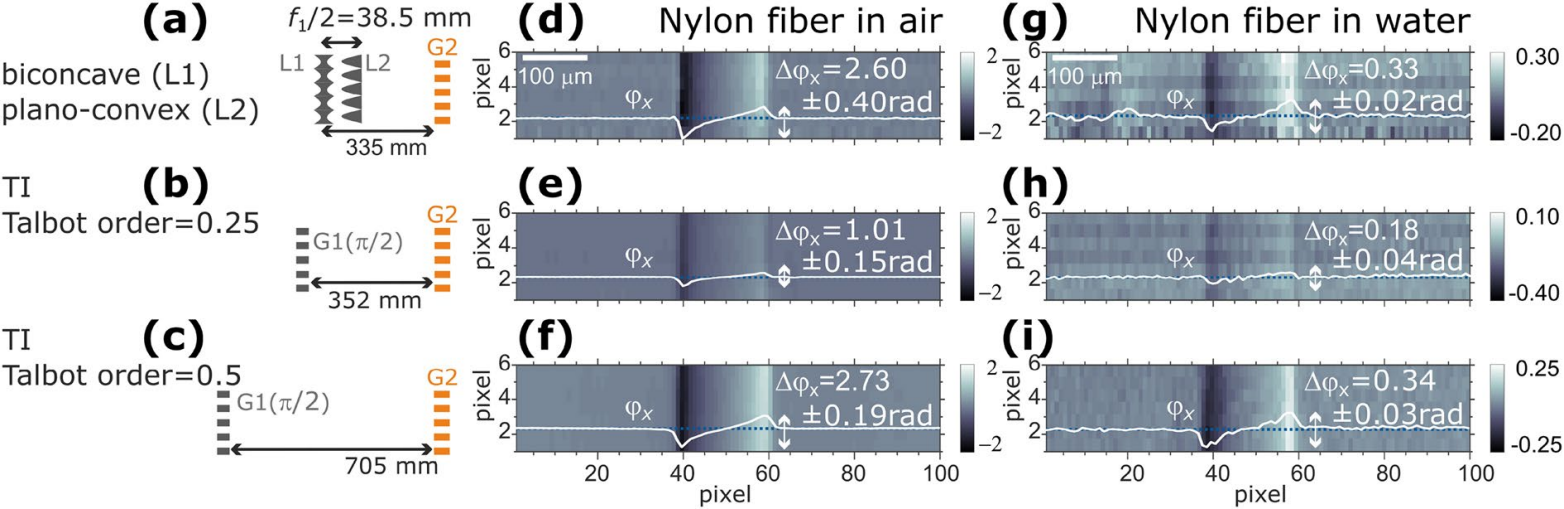
Comparison of differential phase images obtained for a nylon fiber in air and water with (**a**) a biconcave-plano-convex system with a gap of 38.5 mm and an inter-grating distance of 335 mm, (**b**) a conventional Talbot interferometer with a Talbot order of 1/4, and (**c**) that with a Talbot order of 1/2. On each differential phase image, the profile $\phi _x$ across the fiber is depicted. $\Delta \phi _x$ indicates the peak-to-valley value of the profile. The scale bar applies only to the pixels in the horizontal direction. The pixels are enlarged vertically to compensate for the limited FoV.

For an experimental proof-of-concept demonstration of sensitivity enhancement, differential phase images of a nylon fiber with a diameter of 130 $\mu$m were recorded in air and water. The measurements were performed under three different configurations:

- biconcave and plano-convex parabolic lens system with a gap set between $f_1/2$ and $Z_{G2}=Z_\mathrm{{T}}/8$, as shown in Fig. 8a.
- binary $\pi /2$ phase modulating grating with $Z_\mathrm{{G2}}=Z_\mathrm{{T}}/8$, also referred to as Talbot order of 0.25, as shown in Fig. 8b
- binary $\pi /2$ phase modulating grating with $Z_\mathrm{{G2}}=Z_\mathrm{{T}}/4$, also referred to as Talbot order of 0.5, as shown in Fig. 8c

An identical binary absorption grating, described in the previous section, was used for all the experiments. The nylon fiber was placed 100 mm upstream of the biconcave parabolic grating or binary phase grating. For all the experiments, 20-step phase stepping measurements with and without the sample were performed with a step size of 0.5 μm and 30 ms exposure time each (except for the case of the Nylon fiber in water measured with an exposure time of 150 ms, to compensate for the X-ray attenuation by water). The intensities right behind the G2 are registered by a sCMOS camera (Hamamatsu C11440-22CU) coupled with lens system (Hamamatsu AA40) and phosphor screen (P43 10 μm in thickness). The calculation through a phase-stepping technique under all the above configurations was the same as the ones for conventional Talbot interferometry^20^. Differential phase images obtained for the nylon fiber in air are shown in Fig. 8d,e,f, and in water in Figs. 8g,h,i. The differential phase signal decreased when the sample was immersed in water because the difference in the refractive index between the sample and its surroundings is smaller than that in air. In Fig. 8d,e,f,g,h,i, the profiles of the differential phase signal ($\phi _x$) across the nylon fiber are plotted. $\Delta \phi _x$ represent the peak-to-valley values of $\phi _x$ averaged over the length of the fiber within the field of view (FoV). The uncertainties stated for $\Delta \phi _x$ are the standard deviation of $\Delta \phi _x$ along the length of the nylon fiber. We find an enhancement of the phase signal with a factor of 2.6 and 1.8 for the nylon fiber in air and water, respectively, between the proposed configuration and a Talbot interferometer under a Talbot order of 1/4, both of which are nearly comparable in length. It is also demonstrated that the differential phase signal from the Talbot interferometer with a Talbot order of 1/2, whose length of 705 mm is nearly double the others, is comparable to that obtained by the proposed configuration. Thus, by using biconcave and plano-convex lens array pairs, instead of a binary phase grating, the phase signal is enhanced without altering G2 or increasing the inter-grating distance.

## Discussion

In addition to the differential phase signal, grating-based X-ray Talbot interferometry provides both absorption and dark field imaging modalities. The dark field contrast originates from small angle scattering by a sample, which is detectable from the visibility reduction of a phase stepping curve^21^. Recently, the dark field modality has drawn more attention due to its possible benefits in diagnostic medical X-ray imaging^22,23^ and in non-destructive testing, particularly for fiber-composite materials^24^. Since our sensitivity enhancement is based on the angular magnification of the deflected beam from a sample, in addition to the differential phase signal, the dark field signal is enhanced as well, see Supplementary Figure 3.

The proposed configuration can be implemented using a micro-focus source in laboratory like a conventional Talbot interferometer; the periods of L1 and L2 must be designed considering cone-beam illumination. Since this concept is effective with a compact configuration, an X-ray flux density higher than that obtained from a conventional Talbot interferometer will be ensured at the detector position under the same sensitivity condition.

The sensitivity of Talbot interferometry is often attributed to the smallest detectable refraction angle, $\alpha _\mathrm{{min}}$, given by^25–27^

5 $$\begin{aligned} \alpha _\mathrm{{min}} = \frac{P_\mathrm{{G2}}}{2\pi Z_\mathrm{{G2}}} \frac{\sqrt{2}}{V\sqrt{I}}, \end{aligned}$$

where *V* is the visibility (i.e., $|\xi |$) and *I* is the total number of photons detected during a phase stepping measurement. The ratio of $\alpha _\mathrm{{min}}$ for the Talbot interferometry and biconcave-plano-convex systems can be described by

6 $$\begin{aligned} \frac{\alpha _\mathrm{{min,TI}}}{\alpha _\mathrm{{min,CC}}} = M \frac{V_\mathrm{{CC}} \sqrt{I_\mathrm{{CC}}}}{V_\mathrm{{TI}} \sqrt{I_\mathrm{{TI}}}}, \end{aligned}$$

where indices TI and CC correspond to the Talbot interferometry and biconcave-plano-convex system, respectively. For Talbot interferometry using a nickel grating with $\pi /2$ phase modulation at 17 keV, the nickel thickness along the X-ray path was 3 μm, and the parabolic gratings used in this study had a maximum nickel thickness of around 23 $\mu$m, and a minimum of 3 μm, as shown in Supplementary Figure 1. This means that the beam absorption of our set-up was higher than that caused by the Talbot interferometer. In addition, when the plano-convex lens array is positioned closer to the biconcave foci, the converged rays pass through longer length of the plano-convex, meaning even a higher absorption. Therefore, in considering the signal-to-noise ratio of the imaging system, the sensitivity enhancement factor *M* should overcome the reduction of X-ray flux by the parabolic grating set. Under this concept, lighter materials such as Si and polymers would be better suited for parabolic gratings. For higher-energy X-rays, attenuation by the phase grating is less problematic, and therefore the effect of sensitivity enhancement will be more prominent.

During this study, the biconcave and plano-convex gratings were aligned such that the beam propagation direction was parallel to a line connecting the vertices of the concave and convex parabolic curves. The high quality of the structures fabricated by X-ray LIGA ensured the homogeneity of the period and parabolic profile of each micro-lens. The comparison between the measured and simulated propagated wave fields in Fig. 7 shows a very good agreement considering that the simulations were performed for perfectly aligned gratings. If one of the lens arrays is shifted laterally, the effective phase and amplitude modulation of the pair are changed. This results in the alteration of the wave fields and hence the visibility. The effect of the misalignment on the self-image lateral shift enhancement factor (M) remains to be tested in future studies.

The FoV of our biconcave-plano-convex system under plane wave illumination was limited to a nickel structure height of 60 μm, and an array length of 10.24 mm, which was sufficient to confirm the sensitivity enhancement concept. However, the FoV can be increased up to tens of squared centimeters using inclined staircase lens arrays such as the structures demonstrated for super-resolution X-ray transmission microscopy^28^.

In this study, we have mainly focused on finding L1–L2 lens pair configurations in combination with an absorption grating with a period equal to the L1–L2 period and a duty cycle of 0.5. However, the wave fields shown in Fig. 7a,b,c,d indicate the possibility to find a suitable position for an absorption grating with a period smaller than the parabolic grating period and/or a duty cycle smaller than 0.5. These positions are indicated by the local maximas of the intensity variances in Fig. 7.

The lens arrays used in this study were periodic in one direction and generated differential phase images with phase sensitivity in only one direction. It should be noted that this can be extended to two directions by aligning two L1–L2 lens pair sets orthogonally, or more ideally by using paraboloid concave and plano-convex lens arrays. To fabricate the latter, two-photon lithography^29,30^ is expected in the near future.

## Conclusion

In conclusion, by means of simulation studies and experimental validation we have demonstrated the sensitivity enhancement of X-ray Talbot interferometry using parabolic gratings instead of commonly used binary phase gratings. The gratings were fabricated using the deep X-ray lithography, which enables optical side wall quality, and a very good agreement between designed and fabricated structure. With half of the inter-grating distance, the phase signal obtained from our set-up is comparable with a Talbot interferometry using a binary phase grating and the same absorption grating, G2. As an outlook, our concept can be demonstrated with compact X-ray sources. In addition, the feasibility of extending the phase sensitivity to two directions can be implemented by fabricating paraboloid gratings using three-dimensional micro-fabrication techniques, such as two-photon lithography.

## Supplementary Information

Supplementary Information.

## Data Availability

The code used for data analysis as well as for display of the data is available from the corresponding author upon reasonable request. The data sets generated and analyzed during this study are available from the corresponding author upon reasonable request.
