# Supplementary material for: Parabolic gratings enhance the X-ray sensitivity of Talbot interferograms
Source: Sci Rep. 2023 Jun 27;13:9624. doi: 10.1038/s41598-023-36414-8 (PMC10300015; doi:10.1038/s41598-023-36414-8)
Supplement: Supplementary file 1 — Supplementary Information. [file 41598_2023_36414_MOESM1_ESM.pdf]

Supplementary information:

Parabolic gratings enhance the X-ray sensitivity of Talbot interferograms

Supplementary Note 1: extracting parabola's geometry

Figure 1 shows scanning electron microscope (SEM) images obtained for the fabricated parabolic gratings. In order to obtain the structure geometry, sine-cosine Fourier series functions, equation 1, are fitted to the contours extracted from scanning electron microscope images shown in Fig. 1.

$$height\,(x) = a_0 + \sum_{n=1}^{8} \left(a_n \cos\left(n\omega x\right) + b_n \sin\left(n\omega x\right)\right) \tag{1}$$

The ω in equation 1 is $\omega = \frac{2\pi}{P_1}$ with $P_1 = 10\ \mu$m fixed to the period of the structure.

Supplementary Figure 1: parabola’s contours obtained from SEM images

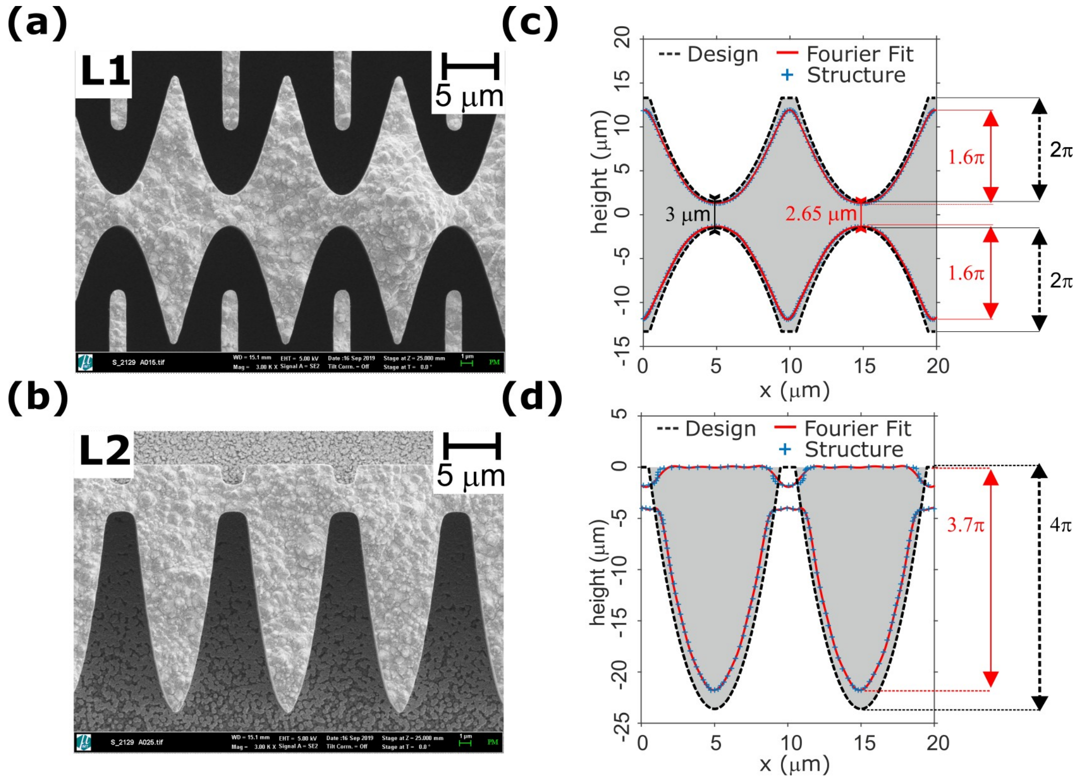

Figure 1. Scanning electron microscope (SEM) images of the nickel parabolic biconcave lens array (L1) and plano-convex lenses (L2) shown in (a) and (b), respectively. The images are taken using Zeiss SUPRA 60VP electron microscope with the stage angle of 0.0°operating at 5.00 kV. The lenses contours are obtained from these images and are depicted in comparison to the designed structure, and the fitted curves for biconcave and plano-convex lenses in (c) and (d), respectively. The maximum phase shift of a monochromatic beam at 17 keV for each parabola design and fabricated structure are shown as well.

Supplementary Figure 2: focal distances of the fabricated parabolic biconcave and plano-convex lenses

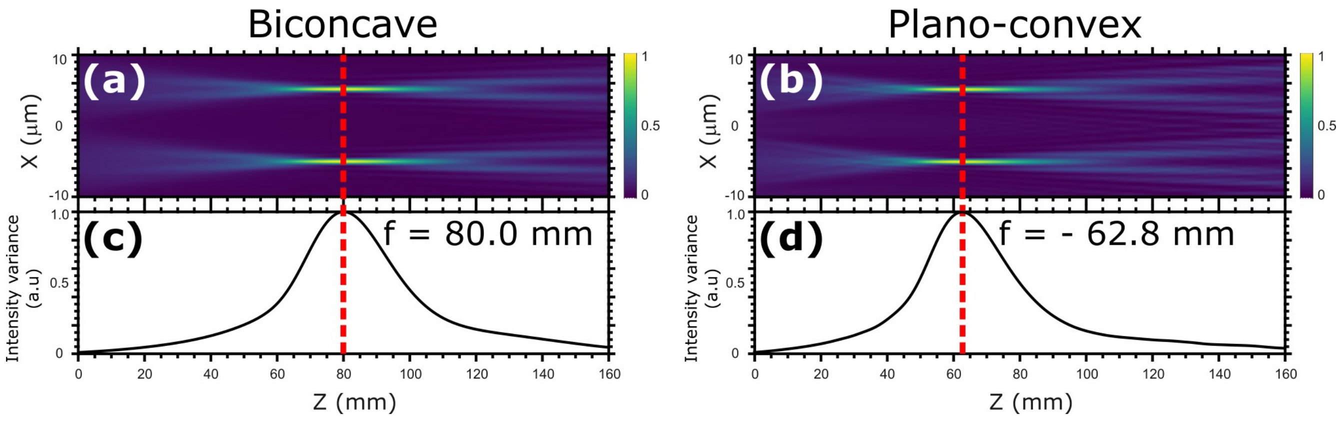

Figure 2. The wave field simulation of nickel biconcave (a) and plano-convex lenses (b) using the fitted curves on the structure contours under plane wave illumination with the energy of 17 keV. The variance peak of the intensities are used for determination of the focal length of each lens and are shown for the biconcave and plano-convex lenses in (c) and (d), respectively. The simulated wave field of the plano-convex lens is performed using the index of refraction decrement of nickel δ_{nickel} multiplied by -1 in order to converge the beam.

Supplementary Note 2: enhancement of the visibility contrast (dark-field)

Figure 3 shows visibility (dark-field) images of a leaf sample in air: Panels (a) - (c) correspond to biconcave-plano-convex system, Talbot interferometry with order 0.25, and Talbot interferometry with order 0.5, respectively. Enhancement of the visibility signal by the proposed configuration is demonstrated.

Supplementary Figure 3: enhancement of the visibility contrast for a Leaf sample

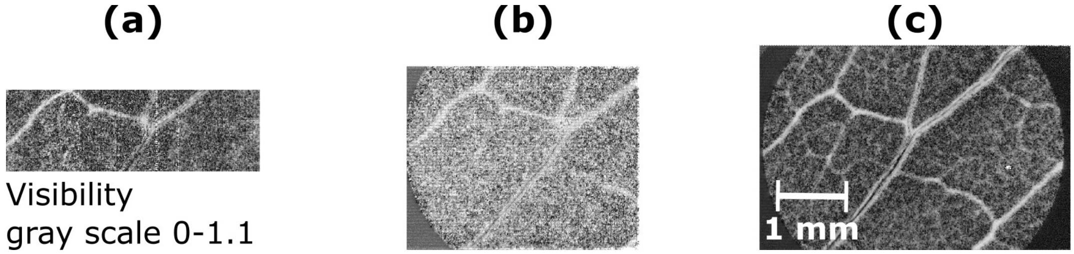

Figure 3. Visibility contrast signal of a Leaf from biconcave-plano-convex system, (a), Talbot interferometry with order 0.25, (b), and Talbot interferometry with order 0.5, (c)
